# Comprehensive Study of Traditional Plant Ground Ivy (*Glechoma hederacea* L.) Grown in Croatia in Terms of Nutritional and Bioactive Composition

**DOI:** 10.3390/foods11050658

**Published:** 2022-02-23

**Authors:** Danijela Šeremet, Stela Jokić, Krunoslav Aladić, Ana Butorac, Marija Lovrić, Ana Jurinjak Tušek, Marko Obranović, Ana Mandura Jarić, Aleksandra Vojvodić Cebin, Klaudija Carović-Stanko, Draženka Komes

**Affiliations:** 1 Faculty of Food Technology and Biotechnology, University of Zagreb, Pierotti St 6, 10000 Zagreb, Croatia; dseremet@pbf.hr (D.Š.); ana.tusek.jurinjak@pbf.unizg.hr (A.J.T.); mobran@pbf.hr (M.O.); amandura@pbf.hr (A.M.J.); avojvodic@pbf.hr (A.V.C.); 2 Faculty of Food Technology, Josip Juraj Strossmayer University of Osijek, Franje Kuhača 20, 31000 Osijek, Croatia; stela.jokic@ptfos.hr (S.J.); krunoslav.aladic@ptfos.hr (K.A.); 3 BICRO BIOCentre, Ltd., Borongajska Cesta 83h, 10000 Zagreb, Croatia; ana.butorac@biocentre.hr (A.B.); marija.lovric@biocentre.hr (M.L.); 4 Faculty of Agriculture, University of Zagreb, Svetošimunska Cesta 25, 10000 Zagreb, Croatia; kcarovic@agr.hr

**Keywords:** extraction, *Glechoma hederacea* L., micro- and macrocomposition, NIR, polyphenols

## Abstract

In the present study, ground ivy was harvested from different natural habitats in Croatia and subjected to screening analysis for nutritional and bioactive composition. To achieve maximum recovery of phenolic compounds, different extraction techniques were investigated—heat-assisted (HAE), microwave-assisted (MAE) and subcritical water (SWE) extraction. Prepared extracts were analysed by spectrophotometric methods, LC-MS/MS and HPLC-PAD methodologies. Results regarding nutritive analyses, conducted using standard AOAC methods, showed the abundance of samples in terms of insoluble dietary fibre, protein, calcium and potassium, while rutin, chlorogenic, cryptochlorogenic, caffeic and rosmarinic acid were the most dominant phenolic compounds. In addition, LC-MS/MS analysis revealed the presence of apigenin and luteolin in glycosylated form. Maximum recovery of target phenolic compounds was achieved with MAE, while SWE led to the formation of new antioxidants, which is commonly known as neoformation. Moreover, efficient prediction of phenolic composition of prepared extracts was achieved using NIR spectroscopy combined with ANN modelling.

## 1. Introduction

Croatia is a country with a rich vascular flora because, despite its small area of 56,594 km^2^, it counts a total of 5536 (4228 species and 1108 subspecies) taxa. In terms of the number of taxa per square unit, Croatia thus ranks third in Europe, after Slovenia and Albania, in floristic richness [1,2]. This exceptional richness is the result of Croatia’s favourable position in four European biogeographical regions (the Alpine, the Continental, the Mediterranean and the Pannonian) and on the border between the Continental and the Mediterranean climatic zones. It was less affected by the Ice Ages and many species have survived as relicts. The great diversity of the land relief (high mountain belts, karstic fields, river valleys and indented coastlines with more than 1000 islands and islets) contributes to the preservation of many plant species and the development of endemic species [3].

Croatia has a long history of plant usage and, until today, many of them have been included in different segments of life. The total number of plants that are in some relationship with humans in Croatia is approximated to be 1144 taxa, mostly used for medicine (25%) and food (12%) [2]. The *Lamiaceae* family is, after *Rosaceae* and *Asteraceae*, the richest in medicinal species in Croatia, as well as in aromatic plants, which are highly appreciated in Croatian traditional cuisine due to their spicy and aromatic properties, such as *Origanum* spp., *Thymus* spp. and *Rosmarinus officinalis* L. [4]. On a global scale, the *Lamiaceae* family is the sixth largest plant family consisting of more than 200 genera and 7000 species, while in Croatia, it makes up 4% of the total vascular flora with 54 subspecies and 172 species [1,5]. *Glechoma hederacea* L., known as ground ivy, is one of them, and although it has been used for generations in folk medicine, up until today, there is a lack of scientifically based information about its biological activity, nutritive and bioactive composition. Among them, antioxidant activity—higher than that of vitamin C and Trolox—as well as the anti-inflammatory and antimutagenic potential of ground ivy, due to the presence of phenolic compounds, have been reported [6,7]. The most common phenolic compounds in plants are flavonoids and phenolic acids, from which antioxidant properties arise from hydroxyl groups in the ring structure and their arrangements [8]. To overcome the limitations of conventionally heat-assisted extraction techniques in terms of loss and degradation of phenolic compounds at elevated temperatures, many innovative techniques have been introduced, such as microwave-assisted extraction and subcritical water extraction. Microwaves are non-ionizing electromagnetic waves between the X-ray and infrared rays in the electromagnetic spectrum. The principle of microwave-assisted extraction is a special heating system that allows homogeneous internal heating of the entire volume of the material, leading to an increase in pressure inside the plant cells, followed by their rupture and release of target compounds [9]. Subcritical water—hot water held at sufficient pressure to maintain the liquid state at a critical temperature between the boiling point of water and the critical point of water—is often used for the extraction of non-polar or organic compounds because the properties of water in its subcritical range correspond to those of organic solvents [10].

The aim of this study was to comprehensively investigate ground ivy harvested from various natural habitats in the continental part of Croatia with a view to evaluating its nutritional and bioactive composition, aiming towards its further possible application in the formulation of functional food as a source of valuable natural compounds. Although ground ivy is a medical plant with a long history of use in folk medicine, it has been insufficiently described with scientifically based information. To achieve maximum extraction efficiency of the compounds of interest—polyphenols—conventional heat-assisted extraction, as well as innovative techniques including microwave-assisted and subcritical water extraction were investigated. To find similarities or differences associated with natural habitats and/or applied extraction techniques, prepared extracts were recorded by near-infrared (NIR) spectroscopy and data were used for principal component analysis (PCA) and artificial neural networks (ANN) modelling. 

## 2. Materials and Methods

### 2.1. Materials and Chemicals

#### 2.1.1. Materials

All samples of ground ivy were harvested in the continental part of Croatia in April 2020: sample G1 was harvested in Lobor (Krapina-Zagorje County), sample G2 in Zagreb (City of Zagreb), sample G3 in Bilogora (Bjelovar-Bilogora County), sample G4 in Sveti Ilija (Varaždin County), sample G5 in Donja Voća (Varaždin County) and samples G6 and G7 in Sikirevci (Brod-Posavina County) (Supplementary Materials, Figures S1 and S2).

#### 2.1.2. Chemicals

Hydrochloric acid, bromocresol green and methyl red indicators, boric acid, Folin–Ciocalteu’s reagent and sodium carbonate were supplied by Kemika (Zagreb, Croatia). Kjeldahl tablets were purchased from CarlRoth (Karsruhe, Germany). An integrated total dietary fibre assay kit was purchased from Megazyme (Wicklow, Ireland). Gallic acid (>97%), rosmarinic acid (97%), caffeic acid (HPLC standard), cryptochlorogenic (>98%), chlorogenic acid (95%), rutin trihydrate (>97%), luteolin (>98%), apigenin (>97%), (S)-6-Methoxy-2,5,7,8-tetramethylchromane-2-carboxylic acid (Trolox), 2,2-Diphenyl-1-picrylhydrazyl (DPPH) and 2,2′-Azino-bis(3-ethylbenzothiazoline-6-sulfonic acid) diammonium salt (ABTS) were purchased from Sigma-Aldrich (St. Louis, MO, USA). Methanol was supplied from Panreac (Barcelona, Spain) and sulfuric acid, hexane, ethanol, formic acid and acetonitrile from Carlo Erba (Val de Reuil, France). Acetone was supplied by Gram-mol d.o.o (Zagreb, Croatia). All chemicals used for experimental procedures were of analytical grade or HPLC grade. 

### 2.2. Methods

#### 2.2.1. Preparation of Plant Materials

The collected aerial parts of the ground ivy were dried at room temperature for 3 days to a dry matter content higher than 90.0% (Table 1). The dried parts were ground and sieved, and a particle size fraction of less than 450 µm was used for further experiments and analyses.

#### 2.2.2. Determination of Micro- and Macrocomposition

The dry matter, crude protein content, crude oil content and crude mineral content were determined according to the AOAC 930.15, AOAC 976.05, AOAC 920.39 and AOAC 942.05 methods, respectively [11,12,13,14]. The analysis of fatty acid composition, using the EN ISO 5509 method, was performed on an Agilent Gas Chromatography 6890 series equipped with an Agilent Inert Mass Selective Detector (Agilent Technologies, Santa Clara, CA, USA) [15]. The high molecular weight insoluble and soluble fibre content was determined using the Integrated Total Dietary Fiber Assay kit according to the AOAC 2011.25 method [16]. The content of micro- and macroelements was analysed using an inductively coupled plasma mass spectrometer (Agilent 7500cx, Agilent Technologies, Tokyo, Japan) [17].

#### 2.2.3. Extraction of Phenolic Compounds

All extraction techniques were performed under optimum parameters determined through mathematical models and 3D surface plots generated in the program Design Expert (version 12, Minneapolis, MN, USA) using response surface methodology and central composite design [18]. Optimization of extraction parameters was performed on sample G3. Distilled water was used as a solvent in all extraction techniques. Conventional heat-assisted extraction (HAE) was performed in an Inko VKZ ERN water bath (Inkolab d.o.o., Zagreb, Croatia) for 10 min at a temperature of 100 °C and with a sample solvent ratio of 1 g/100 mL (*w*/*v*). Microwave-assisted extraction (MAE) was performed in an Ethos Easy advanced microwave digestion system (Milestone, Sorisole, Italy) for 4.93 min at 90 °C and with a sample solvent ratio of 1 g/100 mL (*w*/*v*). Microwave power was held at 900 W until the target temperature was achieved. Subcritical water extraction (SWE) was performed in the system described by Jokić et al. [19] for 5 min at 200 °C and with a sample/solvent ratio of 1 g/100 mL (*w*/*v*). All extractions were followed by centrifugation (9500 rpm, 20 min) and filtration (Whatman^®^ filter papers 4). Extractions were performed in duplicate. The extracts were analysed immediately upon the completion of extractions. 

#### 2.2.4. Determination of Total Phenolic Content (TPC) and Antioxidant Capacity

TPC of prepared extracts was determined following a spectrophotometric method (Genesys 10S UV-VIS Spectrophotometer, Thermo Fisher Scientific, Waltham, MA, USA) described by Singleton and Rossi [20] with some modifications. Reaction mixture contained 7.9 mL of distilled water, 100 µL of diluted extract, 250 µL of Folin–Ciocalteu’s reagent and 1.5 mL of 20% (*w*/*v*) sodium carbonate solution. The blank contained water instead of extract. The absorbance of the reaction mixture was measured at 765 nm after 2 h, while solutions of gallic acid (25–200 µg/mL) were used for construction of a standard calibration curve. The results were expressed as mg gallic acid equivalents/g of the sample’s dry weight (mg GAE/g dw). The measurements were performed in triplicate.

The antioxidant capacity of the prepared extracts was determined using the DPPH and ABTS radical cation decolorization assays [21,22]. The reaction mixture for the DPPH assay contained 3.9 mL of 0.094 mM methanolic DPPH solution and 100 µL of the diluted extract. The blank contained methanol instead of extract. The absorbance of the reaction mixture was measured at 515 nm after 30 min. In the case of the ABTS assay, 7 mM ABTS solution in water and 140 mM potassium peroxodisulfate solution in water were mixed to a final concentration of 2.45 mM potassium peroxodisulfate and left to react for 16 h. Prior to the analysis, the absorbance of the ABTS radical solution was set to 0.700 at 734 nm by diluting it with the ethanol. The reaction mixture consisted of 40 μL of diluted extract and 4.0 mL of the ABTS radical solution. The blank contained ethanol instead of extract. For both assays, the standard calibration curve was constructed using solutions of Trolox (25–200 µg/mL), and the results were expressed as mmol Trolox equivalent/g of the sample’s dry weight (mmol Trolox/g dw). The measurements were performed in triplicate.

#### 2.2.5. HPLC Determination of Individual Phenolic Compounds

The HPLC analysis was performed on an Agilent Series 1200 chromatographic system (Agilent Technologies, Santa Clara, CA, USA) with a Zorbax Extend C18 chromatographic column (4.6 mm × 250 mm, 5 μm i.d.) (Agilent Technologies, Santa Clara, CA, USA) coupled with a Photodiode Array Detector (PAD) (Agilent Technologies, Santa Clara, CA, USA). The mobile phase consisted of two components: (A) 1% (*v*/*v*) formic acid solution in water and (B) 1% (*v*/*v*) formic acid solution in acetonitrile. Elution was performed by increasing the level of component B over time as follows: 0 min—7% B; 5 min—7% B; 45 min—40% B; 47 min—70%; 52 min—70% B at a flow of 1 mL/min. The injection volume was 5 μL and the column temperature was 25 °C. The chromatograms were recorded at 320 and 350 nm. The analysis for all samples was performed in duplicate. All samples were filtered through a 0.45 µm membrane filter (Nylon Membranes, Supelco, Bellefonte, PA, USA) prior to the analysis.

Obtained chromatograms of extracts revealed several peaks that could not be identified due to the lack of appropriate HPLC standards. To identify those, representative extracts of ground ivy (sample G5-MAE and SWE) were subjected to fractionation using an Agilent 1260 Infinity II Analytical-Scale Fraction Collector (Agilent Technologies, Santa Clara, CA, USA) coupled with the previously mentioned Agilent Series 1200 chromatographic system. Peak-based collected fractions (Supplementary Materials, fractions F1, F2, F3 and F4 on Figure S3a,b) were analysed using LC-MS/MS analysis, as described in the following section. 

LC-MS/MS analyses of fraction F1 gave the [M − 1]^−^ ion in negative scan mode at *m*/*z* 353 in accord with a molecular formula C_16_H_18_O_9_. Its molecular ion [M−]^−^ yielded tree peaks at *m/z* 191, 179 and 173 (Supplementary Materials, Figure S4) that suggested, according to the literature [23], the presence of caffeoylquinic acid isomers. Further HPLC-PAD analysis with appropriate HPLC standard revealed it was cryptochlorogenic acid. Further, in the case of fraction F2, LC-MS/MS analyses resulted in the [M − 1]^−^ ion in negative scan mode at *m*/*z* 593, in accordance with the molecular formula C_27_H_30_O_15_, and its molecular ion [M − 1]^−^ yielded one peak at *m*/*z* 285 (Supplementary Materials, Figure S5) that suggested, according to the literature [24,25], it is luteolin-7-*O*-rutinoside. In the absence of a suitable HPLC standard, the identification was further confirmed by subjecting fraction F2 to acid hydrolysis, which should result in the release of luteolin from its conjugated form of glycoside [26]. For this purpose, the collected fraction was evaporated to dryness under nitrogen and dry residue was subjected to acid hydrolysis with 2 M HCl solution (1 h, 80 °C). The cooled hydrolysate was diluted with NaCl solution (5 M) and water to give the 2 M NaCl concentration in a defined volume. Extraction of the liberated luteolin was performed by liquid–liquid extraction with ethyl acetate. Ethyl acetate was completely evaporated under nitrogen, while the dry residue was resuspended in a defined volume of ethanol and subjected to HPLC-PAD analysis. Acid hydrolysis resulted in the release of luteolin in aglycone form, thus confirming the presence of glycosylated luteolin in the ground ivy extract. LC-MS/MS analyses of fraction F3 resulted in the [M + 1]^+^ ion in positive scan mode at *m*/*z* 519 (molecular formula C_24_H_22_O_13_) and its molecular ion [M − 1]^+^ yielded two peaks at *m*/*z* 271 and 433 (Supplementary Materials, Figure S6) that suggested, according to the literature [27], it is apigenin 7-(6″ malonyl glycoside). Identification was further confirmed by applying acid hydrolysis following the same procedure as for the fraction F2. Unlike F1, F2 and F3, F4 remained unidentified and an explanation for this fraction, detected in the SWE extract, is given in the discussion part (Section 3.2). 

Identification of all phenolic compounds was performed by comparing the retention times and characteristic absorption spectrums (190–400 nm) with commercially available standards. Quantification was enabled by establishing calibration curves (2–100 μg/mL). For peaks corresponding to the fractions F2 and F3, quantification was performed using commercial standards of luteolin and apigenin, respectively. 

#### 2.2.6. LC-MS/MS Analysis

The 6460 TripleQuad LC/MS system (Agilent Technologies, Santa Clara, CA, USA) interfaced with an electrospray ion source was used for peak identification. Liquid chromatography separation was performed on an Zorbax Eclipse C18 (2.1 mm × 50 mm, 1.8 μm i.d.) at 30 °C. The isocratic elution was performed with a mobile phase consisting of 0.1% (*v*/*v*) formic acid in 50% acetonitrile aqueous solution (*v*/*v*). The flow was 0.4 mL/min and the injection volume was 1 μL. Total retention time was 5 min. A mass spectrometer was operated in the positive and negative MS scan mode. Ion source parameters were set at gas temperature 250 °C, gas flow 7 L/min, nebulizer 40 psi, sheath gas heater 325 °C, sheath gas flow to 11 L/min, capillary voltage 3500 V and initial fragmentor voltage 200 V. Dominant ions of collected fractions were selected for fragmentation. Fragmentor voltage was optimized for targeted precursors in selected ion monitoring (MS 2 SIM) mode. Finally, for fragmentation, the mass spectrometer was operated in a product ion mode; collision energy was set at 5–40 eV. MS/MS spectra were recorded in negative or positive scan mode, depending on the fraction. 

#### 2.2.7. Near-Infrared Spectroscopy (NIR) 

NIR spectra were gathered in the range 904–1699 nm, using the spectrophotometer NIR-128-1.7-USB/6.25/50 μm (Control Development, Inc., South Bend, IN, USA) with a halogen light source (HL-2000) and with installed CD software Spec32 (Control Development, Inc). Each absorbance spectrum was recorded in triplicate. 

#### 2.2.8. Statistical Analysis 

Principal component analysis (PCA) was employed to compare ground ivy extracts prepared with different extraction methods based on raw NIR spectra in the wavelength ranges 904–928 nm and 1399–1699 nm using Statistica v.10.0 software (StatSoft, Tulsa, OK, USA). Multiple layer perceptron neural networks (MLPs) were developed in Statistica v.10.0 software (StatSoft, Tulsa, USA) for: (i) simultaneous prediction of total phenolic content (TPC) and antioxidant capacity (ABTS and DPPH), and (ii) simultaneous prediction of individual selected phenolic compounds content (chlorogenic acid, cryptochlorogenic acid, caffeic acid, rosmarinic acid and rutin) based on NIR spectra of ground ivy extracts prepared with different extraction techniques. ANN models consisted of an input layer, hidden layer and output layer. The ANN modelling inputs were the coordinates of the first five principal components that contributed to more than 99% of the variability. ANN modelling was carried out on data matrix dimension: (i) 63 × 8 for simultaneous prediction of total phenolic content and antioxidant capacity, and (ii) 63 × 10 simultaneous prediction of individual selected phenolic compounds content (63 rows represent extracts and 8 or 10 columns refer to 5 PCA coordinates (factors) and 3 or 5 column representing analysed results). For the ANN modelling, data were randomly divided at a 70:15:15 ratio for training, testing and validation. Model training was performed using a back error propagation algorithm implemented into Statistica v.10.0 Automated Neural Networks (StatSoft, Tulsa, OK, USA). Proposed ANN model performance was estimated based on R^2^ and Root Mean Squared Error (RMSE) values for training, test and validation and number of neurons in the hidden layer. 

One-way ANOVA and Tukey’s post hoc test were performed in the SPSS Statistics 17.0 software. The differences were considered significant at *p* < 0.05. 

## 3. Results and Discussion 

### 3.1. Macro- and Microcomposition Analysis

Characterization of macro- and microcomposition of ground ivy samples harvested from different natural habitats is presented in Table 1 and Table 2.

Insoluble dietary fibre with a content of 32.26% dw (sample G1)–48.03% dw (sample G4) was the most represented constituent of dry matter in all harvested samples. High content of insoluble dietary fibre can be attributed to a high content of cellulose, a structural polysaccharide that makes up around 50% of all carbon found in plants [28]. Soluble dietary fibre was much less represented—from 4.97% dw in sample G5 to 9.14% dw determined in sample G7. Furthermore, all harvested samples were found to be rich in proteins, especially sample G5 which had a protein content (23.12% dw) almost in the range of grain legumes (24.0–26.1%), which are considered to be rich protein sources [29]. Values of mineral content of the evaluated samples were in a narrow range from 9.20% dw in sample G6 to 10.98% dw in sample G3. Oil was the least represented macrocomponent in all samples, and its content did not exceed more than 2.80% dw (sample G1). As a comparison, a similar macrocomposition, including protein (20.38%), mineral (15.78%) and oil (3.96%) content, was reported for the leaves of ground ivy harvested in South Korea [30].

Profile of saturated, monounsaturated and polyunsaturated fatty acids differed depending on the natural habitat of the plant. However, common to all samples was the dominance of unsaturated fatty acids over saturated ones, with oleic (33.85 and 39.01% fa in samples G2 and G4), linoleic (19.48 and 32.35% fa in samples G3 and G6) and α-linolenic (29.45, 37.92 and 27.15% fa in samples G1, G5 and G7, respectively) acids found in abundance, while among saturated fatty acids, palmitic acid (ranging from 7.14% fa in sample G4 to 13.33% fa in sample G3) was dominant, except in sample G7. Sample G7 showed the greatest diversity of fatty acids in its composition and it is worth pointing out that it was the only sample with identified behenic acid (6.52% fa), and along with sample G1 and G4, the only one with lignoceric acid (13.73% fa), with a significantly (*p* < 0.05) higher content compared to the other two mentioned samples (2.46 and 3.22% fa, respectively). Palmitic, linoleic and α-linolenic acids were previously reported as the main fatty acids in the leaves of different plants of the *Lamiaceae* family [31]. According to Barros et al. [28], a major fatty acid in ground ivy, originating from north-eastern Portugal, was oleic acid (35.12%), followed by α-linolenic acid (27.87%), palmitic acid (12.23%) and linoleic acid (8.15%), which is in good agreement with the results of the present study. 

Content of macro- and microelements is presented in Table 2. Element content in plants may vary depending on the geochemical characteristics of the soil and the plant’s ability to accumulate specific elements [32]. According to the present results, if used as an herbal medicinal product or supplement, ground ivy can serve as a natural source of potassium (K) and calcium (Ca), since all samples showed their abundance (14.49 mg/g dw (G1)–29.80 mg/g dw (G4) and 7.78 mg/g dw (G2)–14.89 mg/g dw (G3), respectively). Potassium intake contributes to a reduction in blood pressure and, thus, reduces the risk of stroke and coronary heart disease and possesses a protective effect on age-related bone loss and reduction in kidney stones, while calcium has been designated as a “super nutrient” due to its role in reducing the risk of osteoporosis, hypertension and possibly colon cancer, as well as other disorders [33,34]. Additionally, if used in the form of a tea infusion, ground ivy still can serve as a rich source of K since it belongs to the group of highly extractable elements [32]. Further, for most samples (G3, G4, G5, G6 and G7), content of macroelements decreased in the following order K > Ca > Mg > P > Al > Fe > Na. A similar order was noticed in samples G1 and G2 with a different order for Mg, Al and P (G1: K > Ca > Mg > Al > P > Fe > Na; G2: K > Ca > P > Mg > Al > Fe > Na). Among investigated microelements, vanadium (V) and chromium (Cr) stand out due to their content, especially in samples G1 (3380 and 3294 µg/kg dw, respectively) and G2 (3793 and 2873 µg/kg dw, respectively). A small amount of vanadium with a common daily intake of 0.01–0.02 mg, taken in via nutrients and drinking water, in the form of oxidovanadium (IV) or -(V) compounds, or more predominant as vanadate H_2_VO_4_^−^, is beneficial for health, while excessive intake can be toxic. Vanadium compounds showed to be useful in treating diabetes, in antitumour and anticancer therapy and as well as in fighting infectious diseases [35]. Medical plants are known as concentrators of Cr and, thus, as well as ground ivy, can serve as food supplements to prevent Cr deficiency that can otherwise cause various cardiovascular disorders, atherosclerosis, endocrine diseases, peripheral neuropathy, etc. [36]. There is no standard regarding the permissible level of metals in medical raw plant material and the World Health Organization only mentions maximum permissible levels for arsenic (1.0 mg/kg), cadmium (0.3 mg/kg) and lead (10 mg/kg) [37]. In the present study, the content of mentioned elements in all harvested samples of ground ivy was below the permissible levels. Regarding other microelements (Cu, Ni, Hg, Mn, Zn, Mo, Co and Sb), their content was within the safe limits and below reference values for toxic effect [38].

### 3.2. Phenolic Profile Analysis

Characterization of ground ivy phenolic profile included determination of TPC, antioxidant capacity by DPPH and ABTS assays and the content of individual phenolic compounds of its extracts. Results are presented in Table 3. 

The highest TPC and antioxidant capacity in all samples of ground ivy were determined in extracts prepared by SWE, with samples G3 and G4 at the minimum and maximum of values range (60.1–101.7 mg GAE/g dw, 0.280–0.584 mmol Trolox/g dw, 0.349–0.650 mmol Trolox/g dw, respectively). Sample G3 was characterized as having the lowest TPC in the case of HAE (40.6 mg GAE/g dw) and MAE (42.9 mg GAE/g dw), while sample G4 was again the richest in TPC using both extraction techniques (87.9 and 95.1 mg GAE/g dw, respectively). The same was noted for antioxidant capacity. Therefore, regarding the TPC and antioxidant capacity of the obtained extracts, the efficiency of the used extraction techniques for all samples can be placed in order: SWE > MAE > HAE. However, the comparison of chromatograms of extracts prepared by MAE (Supplementary Materials, Figure S3a), HAE and SWE (Supplementary Materials, Figure S3b) revealed significant changes in phenolic profile. In extracts obtained by HAE and MAE, predominant phenolic compounds were phenolic acids—chlorogenic, cryptochlorogenic, caffeic and rosmarinic acid and rutin, as well—from the group of flavonoids, while their content in SWE extracts was significantly (*p* < 0.05) lower (Table 3). A dominant peak (Supplementary Materials, Figure S3b, peak marked as “F4”), detected only in the SWE extracts, was subjected to LC-MS/MS analysis (procedure as described in Section 2.2.5 and Section 2.2.6) and gave the [M − 1]^−^ ion at *m*/*z* 239 as a base peak that yielded two peaks at *m*/*z* 147 and 193 in MS/MS (Supplementary Materials, Figure S7), but these data could not be used for successful identification because the required information is lacking in the available literature. Although the molecular formula and structure of the dominant peak in SWE extracts remained rather unclear and unknown, it is evident that SWE resulted in the formation of a new antioxidant. This process, called neoformation, means that compounds can be found in the extract that are not present in the natural matrix before extraction [39]. Due to the applied high temperature (200 °C) during SWE, the new antioxidant could have been formed as a degradation product that maintained its phenol structure and, thus, antioxidant capacity. The second possibility could be that the “unknown” peak originated from Maillard reactions and caramelization—processes that occurred during SWE—and also possesses high antioxidant capacity [40].

MAE resulted in the highest recovery of target phenolic compounds—rutin, chlorogenic, cryptochlorogenic, caffeic and rosmarinic acid—that were present in all samples (Table 3). If examining one extraction technique, for example MAE, through all samples, it is obvious that the phenolic profile of ground ivy differed according to the natural habitat—in samples G1, G2 and G6, the predominant phenolic compound was chlorogenic acid (6.10, 7.15 and 4.98 mg/g dw, respectively); in samples G2 and G7 rutin (8.83 and 3.87 mg/g dw, respectively), while in G3, G4 and G5 rosmarinic acid (5.56, 18.69 and 4.14 dw, respectively). Similar results, including the dominance of rosmarinic acid, and with the presence of caffeic and chlorogenic acid in ground ivy, were reported by Chou et al. [7] and Belščak-Cvitanović et al. [41]. Additionally, LC-MS/MS analysis revealed the presence of flavonoids apigenin and luteolin in glycosylated forms (Section 2.2.5): apigenin 7-(6″ malonyl glycoside) and luteolin-7-*O*-rutinoside with the highest content in samples G2 (0.90 mg/g dw) and G5 (0.68 mg/g dw), respectively. It is noteworthy to point out the unsuitability of SWE for the extraction of apigenin 7-(6″ malonyl glycoside), since it was not identified in any of the SWE extracts. From the group of flavonoids, Chou et al. [7] reported the presence of daidzein, genistin and genistein in ground ivy, while Kikuchi et al. [42] reported several phenol glycosides such as apigenin 7-*O*-neohesperidoside, chrysoeriol 7-*O*-neohesperidoside and (+)-pinoresinol 4,4′-bis-*O-β*-d-glucopyranoside in ground ivy.

### 3.3. NIR Spectroscopy, PCA and ANN Modelling 

**Figure 1:**
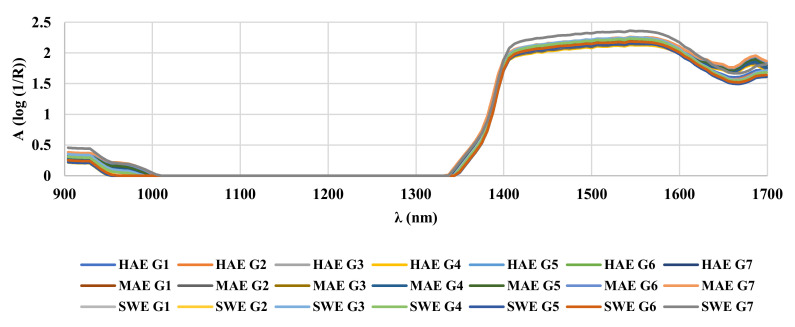
NIR spectra of ground ivy extracts.

NIR belongs to the electromagnetic spectrum between visible light and mid-infrared light (780–2500 nm) and the basic principle of NIR spectroscopy is the irradiation of the sample with NIR light and recording the reflected or transmitted radiation. NIR records the overtones and combination band information of the fundamental vibration of a single chemical bond in a molecule [43]. In the present study, the applied NIR was in the range 904–1699 nm, in which the sample absorbs the light with frequencies matching characteristic vibrations generated from C-H, O-H, N-H and C=O [44], and it was sufficient to achieve data differentiation between the extracts. As can be seen from Figure 1, NIR spectra of extracts of ground ivy harvested from different natural habitats showed high similarity, but differences in the intensity of some characteristic bands were noted, probably influenced by variations in the phenolic profile depending on the natural habitat and/or extraction technique. Significant differences in the NIR spectra between extracts were observed from 904 to 928 nm and from 1399 to 1699 nm and these data were afterward used for the PCA. Shifts in that wavelength range indicated changes in the third and second overtone of the C-H and O-H relations, also related to the hydroxyl group bound directly to an aromatic hydrocarbon [45].

**Figure 2:**
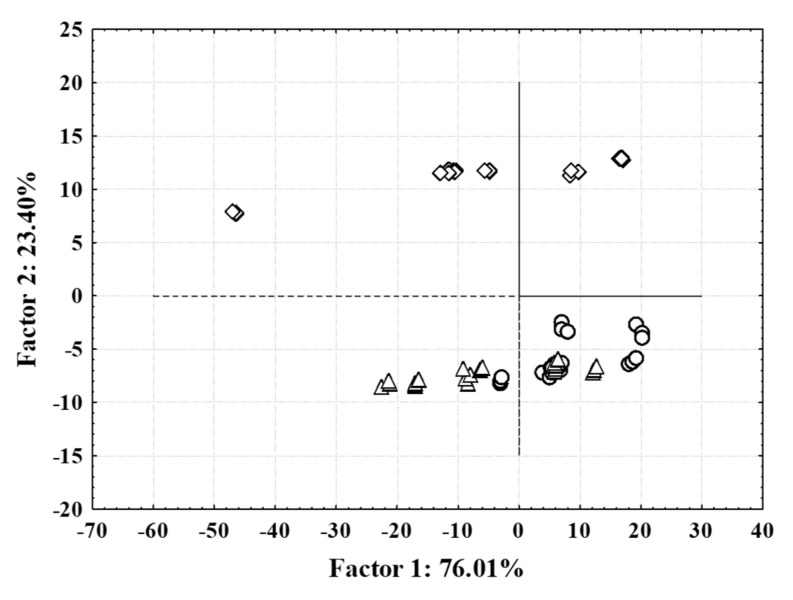
Principal component analysis of NIR spectra of ground ivy extracts: (○) conventionally heat-assisted extraction; (△) microwave-assisted extraction; (◇) subcritical water extraction.

To find similarities or differences associated with natural habitats of ground ivy and applied extraction techniques, PCA was performed with all extracts using the NIR spectrum. PCA derived the important information from the obtained NIR spectrum of extracts and expressed it as a set of new orthogonal variables—principal components. A scatter plot of the first principal component vs. second principal component is presented in Figure 2 with related loadings.

First two principal components explained 99.41% of the total variance in the observed dataset. Results showed grouping of the samples in two specific groups based on the applied extraction technique (HAE, MAE and SWE). One group was formed by extracts prepared using HAE and MAE and the second group was formed by extracts prepared by SWE. The results are in accordance with the HPLC analysis of individual phenolic compounds, since, in all extracts prepared by HAE and MAE, the same phenolic compounds were identified and quantified as dominant, while SWE yielded extracts with a more diverse phenolic profile, as already explained in the previous section. Similar results were reported by Valinger et al. [46] where an efficient grouping of industrial hemp extracts based on combined UV–vis–NIR spectra was achieved according to extraction solvent concentration. 

Furthermore, the applicability of an ANN model for simultaneous prediction of total phenolic content, antioxidant capacity and individual selected phenolic compounds of prepared extracts based on NIR spectra was analysed. The ANN model input was chosen from the coordinates of the first five components acquired by PCA analysis, which contributed more than 99% of the overall variance. Table 4 shows the properties of several of the developed artificial neural networks for both analysed groups of model output. 

**Figure 3:**
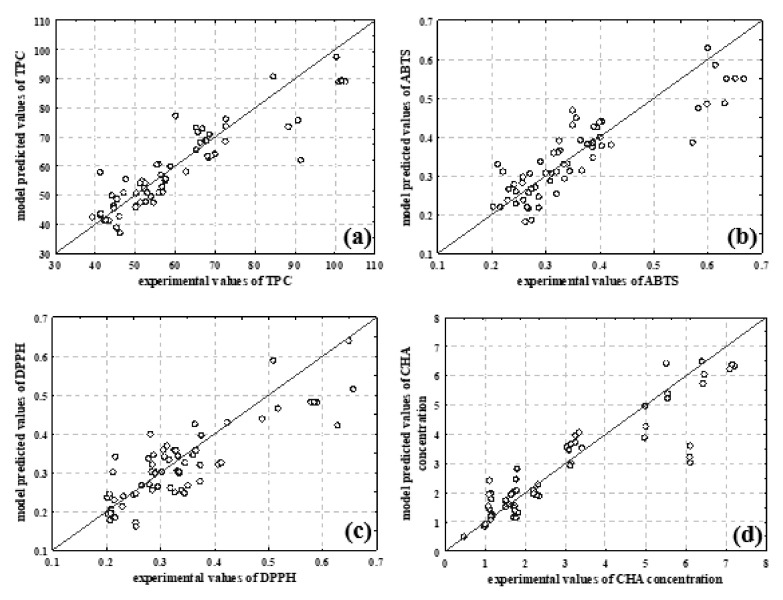

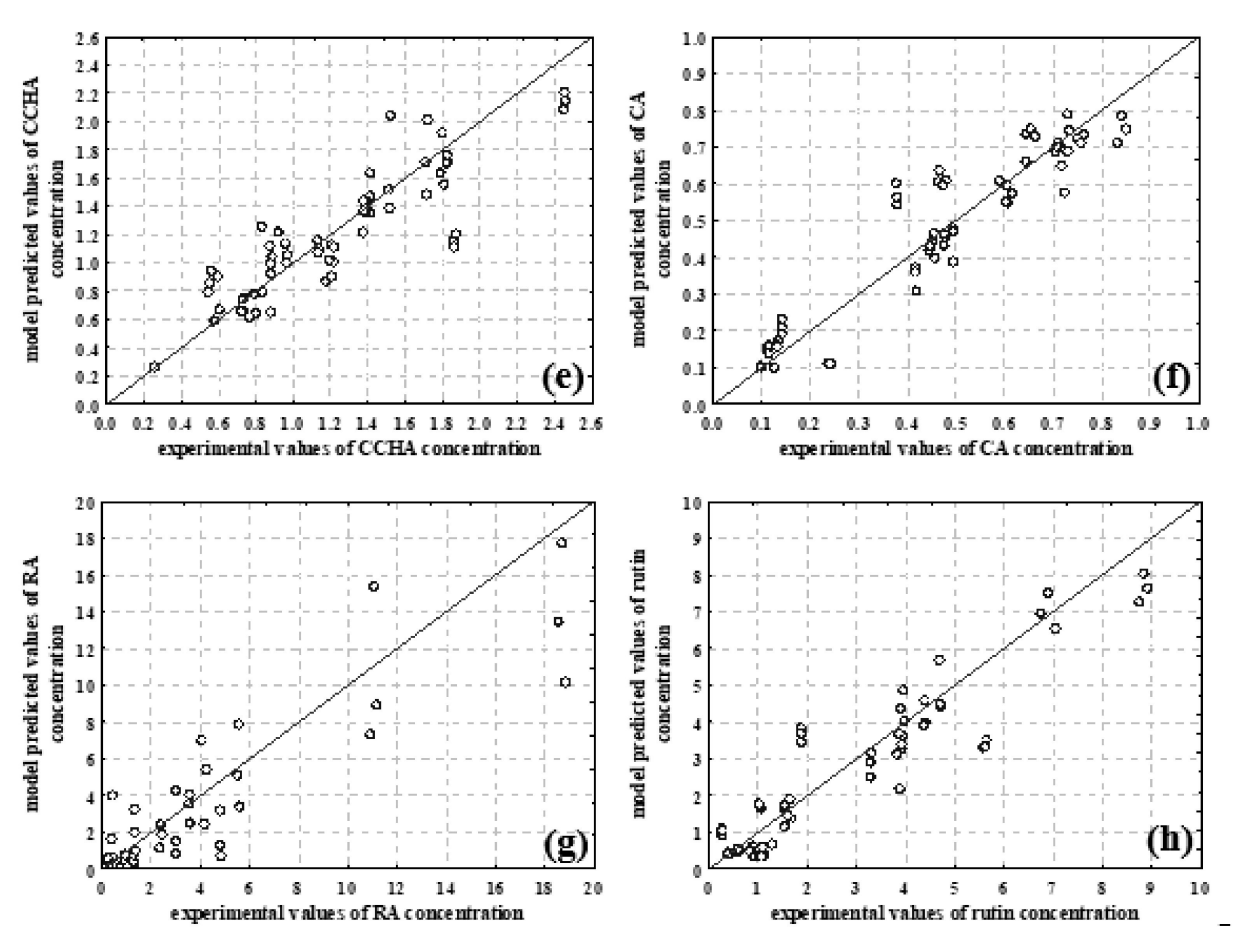
Comparison between experimental data and model predicted data for (**a**) TPC, (**b**) ABTS-measured antioxidant capacity, (**c**) DPPH-measured antioxidant capacity, (**d**) CHA—chlorogenic acid concentration, (**e**) cCHA—cryptochlorogenic acid concentration, (**f**) CA—caffeic acid concentration, (**g**) RA—rosmarinic acid concentration, and (**h**) rutin concentration.

Because the coefficients of determination at all three stages were higher than 0.78 and the root mean square errors for validation of the selected models were low (RMSE < 0.07), the results showed that all five of the selected ANNs provided good agreement between experimental values and model predicted values at the level of learning, testing and validation. The multilayer perceptron network MLP 5-8-3 was selected as the optimum for simulations prediction of TPC, DPPH and ABTS (*R*^2^_training_ = 0.8862, RMSE_training_ = 0.0252, *R*^2^_test_ = 0.8895, RMSE_test_ = 0.0216, *R*^2^_validation_ = 0.8883, RMSE_validation_ = 0.0332). The selected network had five neurons in the input layer, eight neurons in the hidden layer and three neurons in the output layer. Furthermore, the hidden activation function was Exponential and the output activation function was Tanh. As given in Table 5 and Figure 3a–c, the selected MPL 5-8-3 described analysed bioactive properties of the prepared extracts accurately. 

Results (Table 4) show that the best agreement between experimental data and ANN model data was obtained for TPC values (*R*^2^_training_ = 0.9207, *R*^2^_test_ = 0.9079, *R*^2^_validation_ = 0.8928). Moreover, MLP 5-8-5 (*R*^2^_training_ = 0.9433, RMSE_training_ = 0.0189, *R*^2^_test_ = 0.8895, RMSE_test_ = 0.0379, *R*^2^_validation_ = 0.8884, RMSE_validation_ =0.05155) with Logistic function was a hidden activation function, and an output activation function was chosen for prediction of the content of individual selected phenolic compounds (Table 5). Based on the results (Table 5, Figure 3d–h), the best developed ANN model most precisely predicted rutin content (*R*^2^_validation_ = 0.9049), followed by caffeic acid content (*R*^2^_validation_ = 0.8999) and chlorogenic acid content (*R*^2^_validation_ = 0.8826). The presented result showed that NIR spectroscopy combined with ANN modelling can be effectively used to describe phenolic extraction from plant material, as previously described in the literature [46,47]. Rapid and high-throughput analysis, on-site capability, chemical specificity, and minimal sample preparation are all advantages of NIR that have significant potential in plant extracts analysis [48].

## 4. Conclusions

Nutritional and bioactive composition of ground ivy differed depending on the natural habitat, but all samples proved to be valuable sources of insoluble dietary fibre, proteins, calcium and potassium. Besides natural habitat, the bioactive content of ground ivy extracts was greatly influenced by the applied extraction technique. Subcritical water extraction resulted in the formation of new antioxidants and requires further research, but in the present study, microwave-assisted extraction resulted in the highest recovery of target phenolic compounds. The obtained results showed that ground ivy is a plant with a diverse and valuable nutritional and bioactive composition, thus representing a great source of natural compounds with the potential to be used in the formulation of functional foods.

## Figures and Tables

**Table 1:** Nutritional composition of ground ivy harvested from different natural habitats.

Sample	G1	G2	G3	G4	G5	G6	G7
Dry matter (%)	91.13 ± 0.10 ^abc^	91.86 ± 0.06	90.96 ± 0.03 ^a^	91.33 ± 0.14 ^bde^	91.27 ± 0.05 ^cdf^	91.45 ± 0.01 ^ef^	93.15 ± 0.12
Crude protein content (% dw *)	15.63 ± 0.13 ^abc^	14.75 ± 0.23 ^d^	15.61 ± 0.20 ^adef^	17.74 ± 0.17	23.13 ± 0.33	15.86 ± 0.27 ^beg^	16.16 ± 0.16 ^cfg^
Crude oil content (% dw)	2.80 ± 0.54 ^abcde^	2.05 ± 0.07 ^afghij^	1.10 ± 0.20 ^fk^	2.63 ± 0.71 ^bglmn^	1.86 ± 0.15 ^chklop^	2.39 ± 0.10 ^dimor^	2.30 ± 0.06 ^ejnpr^
Myristic acid, C14:0 (% fa *)	0.18 ± 0.02 ^a^	nd	4.53 ± 0.48	0.18 ± 0.05 ^a^	nd	nd	1.25 ± 0.03
Palmitic acid, C16:0 (% fa)	7.47 ± 0.09 ^abc^	7.70 ± 0.23 ^adef^	13.33 ± 2.28 ^g^	7.14 ± 0.14 ^bdh^	10.24 ± 0.82 ^eij^	11.68 ± 0.86 ^gi^	7.68 ± 0.42 ^cfhj^
Palmitoleic acid, C16:1 (% fa)	0.93 ± 0.03	nd	nd	0.14 ± 0.01	nd	nd	1.07 ± 0.03
Heptadecanoic acid, C17:0 (% fa)	0.19 ± 0.01	nd	nd	0.07 ± 0.01	nd	nd	0.39 ± 0.02
Stearic acid, C18:0 (% fa)	2.28 ± 0.50 ^ab^	4.40 ± 1.25 ^c^	15.46 ± 0.50 ^d^	1.59 ± 0.00 ^ae^	16.33 ± 0.30 ^d^	5.89 ± 0.05 ^c^	1.88 ± 0.05 ^be^
Oleic acid, C18:1 (% fa)	28.88 ± 0.31	33.85 ± 1.76	18.19 ± 0.81	39.01 ± 0.10	nd	21.64 ± 0.03	8.70 ± 0.41
Linoleic acid, C18:2 (% fa)	24.51 ± 0.12 ^a^	22.63 ± 0.22 ^b^	19.48 ± 0.44	21.71 ± 0.50 ^b^	24.95 ± 0.50 ^a^	32.35 ± 0.43	14.92 ± 0.29
α-linolenic acid, C18:3 n3 (% fa)	29.45 ± 0.14	24.13 ± 0.81	18.25 ± 0.55	20.27 ± 0.51 ^a^	37.92 ± 1.44	21.96 ± 0.14 ^a^	27.15 ± 0.38
Arachidic acid, C20:0 (% fa)	0.34 ± 0.16 ^a^	nd	nd	0.25 ± 0.01 ^a^	nd	2.15 ± 0.13	0.63 ± 0.04
Behenic acid, C22:0 (% fa)	nd	nd	nd	nd	nd	nd	6.52 ± 0.19
Lignoceric acid, C24:0 (% fa)	2.46 ± 0.13	nd	nd	3.22 ± 0.42	nd	nd	13.73 ± 0.21
Crude mineral content (% dw)	9.76 ± 0.09 ^abcd^	9.59 ± 0.10 ^aefg^	10.98 ± 0.44 ^h^	10.95 ± 0.23 ^h^	10.02 ± 0.13 ^bei^	9.20 ± 0.02 ^cf^	9.97 ± 0.40 ^dgi^
Total dietary fibre (% dw)	37.74 ± 2.11 ^a^	43.14 ± 1.15 ^bcd^	46.65 ± 1.19 ^bef^	55.23 ± 3.42	41.44 ± 0.65 ^acg^	44.05 ± 1.08 ^deg^	49.76 ± 1.10 ^f^
Insoluble dietary fibre (% dw)	32.26 ± 0.55 ^a^	34.14 ± 0.65 ^a^	41.50 ± 0.82 ^b^	48.03 ± 0.18	36.47 ± 0.82 ^c^	37.03 ± 1.15 ^c^	40.61 ± 0.85 ^b^
Soluble dietary fibre (% dw)	5.48 ± 0.74 ^abc^	9.00 ± 0.89 ^d^	5.15 ± 0.43 ^ae^	7.19 ± 0.72 ^f^	4.97 ± 0.38 ^be^	7.02 ± 0.40 ^cf^	9.14 ± 0.26 ^d^

* dw—dry weight of the sample; fa—fatty acids; nd—not detected. Means in the same row denoted with the same superscript letters are not significantly different (*p* > 0.05).

**Table 2:** Macro- and microelement content in ground ivy harvested from different natural habitats.

Sample	G1	G2	G3	G4	G5	G6	G7
Macroelements (mg/kg dw *)							
Na	58 ± 2 ^ab^	98 ± 4	72 ± 2 ^c^	81 ± 1 ^d^	62 ± 3 ^ae^	73 ± 7 ^cd^	62 ± 3 ^be^
Mg	3289 ± 85 ^a^	2044 ± 19	5500 ± 81	2835 ± 30	6671 ± 139	3358 ± 26 ^a^	3695 ± 32
Al	1862 ± 94	1612 ± 54	669 ± 15 ^ab^	693 ± 56 ^cd^	219 ± 15	667 ± 104 ^ac^	668 ± 74 ^bd^
K	14,448 ± 355	20,289 ± 294	28,373 ± 568 ^ab^	29,794 ± 340	27,528 ± 303 ^ac^	26,020 ± 259	27,800 ± 445 ^bc^
Ca	9763 ± 198	7783 ± 104	14,800 ± 219	12,254 ± 125 ^a^	13,115 ± 277	10,803 ± 96	12,072 ± 78 ^a^
Fe	1430 ± 62 ^a^	1322 ± 62 ^a^	498 ± 16 ^bcd^	616 ± 69 ^b^	187 ± 9	476 ± 78 ^ce^	443 ± 44 ^de^
P	1705 ± 31	2187 ± 34	2409 ± 52	2065 ± 22	2961 ± 45	2877 ± 25 ^a^	2822 ± 23 ^a^
Microelements (µg/kg dw)							
V	3380 ± 96	3793 ± 130	1103 ± 17 ^ab^	1474 ± 131	383 ± 23	1132 ± 185 ^ac^	1058 ± 112 ^bc^
Mn	48 ± 2 ^a^	59 ± 1 ^b^	214 ± 3	57 ± 2 ^b^	185 ± 7	43 ± 1 ^a^	66 ± 1
Cr	3294 ± 104	2873 ± 77	2073 ± 16 ^a^	1980 ± 175 ^a^	368 ± 16	1547 ± 88	1118 ± 140
Co	521 ± 18	588 ± 4	267 ± 5	314 ± 29	129 ± 9	165 ± 17 ^a^	177 ± 13 ^a^
Ni	2029 ± 67 ^a^	1635 ± 17 ^bc^	2008 ± 9 ^a^	1668 ± 109 ^b^	1549 ± 54 ^c^	3284 ± 162	2651 ± 68
Cu	8 ± 0	14 ± 0	9 ± 0 ^a^	8.74 ± 0 ^a^	9.90 ± 0 ^b^	11 ± 1	9.86 ± 0 ^b^
Zn	26 ± 0	29 ± 0 ^a^	53 ± 1	30 ± 1 ^a^	63 ± 1	55 ± 1	44 ± 0
As	369 ± 6	460 ± 5	209 ± 3 ^a^	192 ± 55 ^ab^	62 ± 3	147 ± 22 ^bc^	126 ± 13 ^c^
Se	19 ± 1	24 ± 1 ^abc^	25 ± 3 ^ade^	14 ± 1 ^f^	13 ± 1 ^f^	26 ± 2 ^bdg^	25 ± 1 ^ceg^
Mo	1228 ± 21 ^a^	1355 ± 23	337 ± 2	1231 ± 16 ^a^	831 ± 10	637 ± 50	481 ± 0
Cd	14 ± 0 ^abc^	19 ± 1 ^def^	88 ± 5	11 ± 1 ^a^	19 ± 1 ^bdg^	18 ± 1 ^ceg^	23 ± 1 ^f^
Sn	106 ± 6 ^a^	114 ± 3 ^a^	42 ± 1 ^bcd^	50 ± 0 ^bef^	42 ± 1 ^ceg^	75 ± 16	44 ± 5 ^dfg^
Sb	38 ± 1	53 ± 1	19 ± 1 ^ab^	23 ± 2	19 ± 1 ^ac^	27 ± 2	19 ± 1 ^bc^
Hg	18 ± 2 ^ab^	30 ± 1	18 ± 1 ^ac^	12 ± 0 ^de^	13 ± 1 ^d^	16 ± 1 ^bcf^	14 ± 0 ^ef^
TI	24 ± 0	18 ± 0	15 ± 0	6 ± 0 ^ab^	5 ± 0 ^a^	8 ± 1 ^c^	7 ± 1 ^bc^
Pb	957 ± 3	1323 ± 17	418 ± 5 ^a^	374 ± 52 ^bc^	261 ± 8 ^d^	471 ± 71 ^ab^	327 ± 38 ^cd^

* dw—dry weight of the sample. Means in the same row denoted with the same superscript letters are not significantly different (*p* > 0.05).

**Table 3:** Total phenolic content (TPC), antioxidant capacity and content of individual phenolic compounds in ground ivy.

Extraction Technique	Sample	Total Phenolic Content (mg GAE/g dw *)	Antioxidant Capacity (mmol Trolox/g dw)		Phenolic Acids (mg/g dw)				Flavonoids (mg/g dw)		
			DPPH	ABTS	Chlorogenic Acid	Crypto-Chlorogenic Acid	Caffeic Acid	Rosmarinic Acid	Rutin	Apigenin 7-(6″ Malonyl Glycoside) ^1^	Luteolin-7-*O*-Rutinoside ^2^
HAE	G1	50.78 ± 0.67 ^a^	0.232 ± 0.014	0.239 ± 0.013	5.53 ± 0.03	1.51 ± 0.01	0.48 ± 0.02 ^f^	0.96 ± 0.06	4.69 ± 0.02	0.31 ± 0.00	0.54 ± 0.00
MAE		51.63 ± 0.66 ^a^	0.254 ± 0.005	0.257 ± 0.027	6.10 ± 0.01	1.86 ± 0.01	0.47 ± 0.01 ^f^	1.33 ± 0.02	5.61 ± 0.03	0.26 ± 0.00	0.61 ± 0.01
SWE		72.62 ± 0.09	0.304 ± 0.003	0.356 ± 0.007	1.73 ± 0.00	1.39 ± 0.01	0.10 ± 0.00	0.17 ± 0.03	1.01 ± 0.00	nd	0.07 ± 0.00
HAE	G2	56.20 ± 2.75 ^b^	0.266 ± 0.010	0.322 ± 0.003	6.42 ± 0.03	1.71 ± 0.01	0.73 ± 0.00	0.75 ± 0.00	6.89 ± 0.15	0.88 ± 0.00	0.42 ± 0.00
MAE		56.10 ± 0.40 ^b^	0.324 ± 0.007 ^d^	0.336 ± 0.008	7.15 ± 0.06	2.45 ± 0.01	0.65 ± 0.01	3.00 ± 0.01	8.83 ± 0.09	0.90 ± 0.00	0.51 ± 0.00
SWE		66.82 ± 1.66	0.329 ± 0.004 ^d^	0.399 ± 0.004	2.20 ± 0.00	1.82 ± 0.00	0.13 ± 0.00	0.17 ± 0.00	1.09 ± 0.21	nd	0.05 ± 0.00
HAE	G3	40.60 ± 0.93 ^c^	0.209 ± 0.006	0.209 ± 0.005	1.13 ± 0.02 ^e^	0.55 ± 0.01	0.38 ± 0.00	4.80 ± 0.02	1.53 ± 0.01	nd	nd
MAE		42.88 ± 1.41 ^c^	0.243 ± 0.007	0.263 ± 0.004	1.10 ± 0.02 ^e^	0.59 ± 0.01	0.45 ± 0.00	5.56 ± 0.04	1.64 ± 0.03	nd	nd
SWE		60.09 ± 2.55	0.280 ± 0.004	0.349 ± 0.017	0.47 ± 0.00	0.26 ± 0.00	0.14 ± 0.00	0.40 ± 0.00	0.40 ± 0.00	nd	nd
HAE	G4	87.87 ± 2.58	0.504 ± 0.012	0.609 ± 0.020	1.65 ± 0.05	0.83 ± 0.04	0.84 ± 0.01	11.03 ± 0.15	3.89 ± 0.02	0.27 ± 0.00	0.35 ± 0.01
MAE		94.10 ± 4.37	0.554 ± 0.012	0.589 ± 0.013	1.78 ± 0.01	0.96 ± 0.01	0.60 ± 0.00	18.69 ± 0.14	4.38 ± 0.03	0.26 ± 0.00	0.40 ± 0.00
SWE		101.72 ± 0.79	0.584 ± 0.006	0.650 ± 0.016	0.99 ± 0.02	0.73 ± 0.01	0.24 ± 0.00	1.07 ± 0.00	1.01 ± 0.12	nd	0.03 ± 0.00
HAE	G5	44.79 ± 2.00	0.246 ± 0.011	0.276 ± 0.007	1.76 ± 0.06	0.77 ± 0.03	0.47 ± 0.02 ^g^	0.94 ± 0.33	0.58 ± 0.01	0.79 ± 0.02	0.43 ± 0.01
MAE		54.31 ± 0.95	0.284 ± 0.001	0.317 ± 0.006	2.31 ± 0.03	1.19 ± 0.02	0.47 ± 0.02 ^g^	4.14 ± 0.08	1.03 ± 0.02	0.81 ± 0.00	0.68 ± 0.00
SWE		69.00 ± 0.79	0.334 ± 0.001	0.403 ± 0.017	1.17 ± 0.00	0.88 ± 0.00	0.42 ± 0.00	0.24 ± 0.00	0.26 ± 0.00	nd	0.13 ± 0.00
HAE	G6	44.86 ± 0.82	0.208 ± 0.006	0.248 ± 0.006	3.14 ± 0.11	0.87 ± 0.05	0.62 ± 0.03	0.40 ± 0.03	1.88 ± 0.00	0.35 ± 0.01	0.35 ± 0.00
MAE		55.72 ± 1.35	0.273 ± 0.002	0.319 ± 0.011	4.98 ± 0.01	1.80 ± 0.01	0.71 ± 0.00	3.54 ± 0.01	3.95 ± 0.02	0.40 ± 0.00	0.39 ± 0.00
SWE		65.63 ± 0.53	0.286 ± 0.001	0.388 ± 0.012	1.52 ± 0.00	1.38 ± 0.01	0.11 ± 0.00	0.21 ± 0.00	0.82 ± 0.00	nd	0.08 ± 0.00
HAE	G7	44.97 ± 1.18	0.206 ± 0.001	0.256 ± 0.011	3.12 ± 0.02	1.21 ± 0.01	0.72 ± 0.01	1.27 ± 0.00	3.29 ± 0.02	0.08 ± 0.00	0.34 ± 0.00
MAE		49.95 ± 1.85	0.225 ± 0.003	0.292 ± 0.005	3.33 ± 0.07	1.41 ± 0.00	0.76 ± 0.01	2.37 ± 0.06	3.87 ± 0.05	0.11 ± 0.00	0.39 ± 0.00
SWE		67.87 ± 0.17	0.289 ± 0.006	0.387 ± 0.000	1.14 ± 0.00	1.13 ± 0.00	0.13 ± 0.00	0.12 ± 0.00	1.07 ± 0.00	nd	0.09 ± 0.00

* dw—dry weight of the sample; GAE—gallic acid equivalents; ^1^ expressed as apigenin; ^2^ expressed as luteolin; HAE—heat-assisted extraction; MAE—microwave-assisted extraction; SWE—subcritical water extraction; nd—not detected. Means in the same column within the same sample denoted with the same superscript letters are not significantly different (*p* > 0.05).

**Table 4:** Architecture of ANNs developed for prediction of (i) total phenolic content and antioxidant capacity, and (ii) content of individual selected phenolic compounds in ground ivy extracts based on NIR spectra (selected networks are marked bold).

Output	Network Structure	Training Perf. Training Error	Test Perf. Test Error	Validation Perf. Validation Error	Hidden Activation Function	Output Activation Function
Total phenolic content and antioxidant capacity	MLP 5-6-3	0.9475	0.8858	0.8780	Tanh	Exponential
		0.0113	0.0144	0.0357		
	MLP 5-8-3	0.8801	0.8787	0.8726	Exponential	Exponential
		0.0341	0.0416	0.0497		
	**MLP 5-8-3**	**0.8862**	**0.8895**	**0.8883**	**Exponential**	**Tanh**
		**0.0252**	**0.0216**	**0.0332**		
	MLP 5-11-3	0.9567	0.9400	0.9153	Logistic	Exponential
		0.0093	0.0076	0.0256		
	MLP 5-11-3	0.9220	0.9231	0.7813	Exponential	Identity
		0.0162	0.0199	0.0582		
Content of individual selected phenolic compounds	**MLP 5-8-5**	**0.9433**	**0.8909**	**0.8884**	**Logistic**	**Logistic**
		**0.0189**	**0.0379**	**0.05155**		
	MLP 5-9-5	0.9309	0.8899	0.8604	Exponential	Identity
		0.0223	0.0444	0.0562		
	MLP 5-10-5	0.9439	0.8851	0.8625	Logistic	Exponential
		0.0178	0.0380	0.0585		
	MLP 5-10-5	0.9341	0.8704	0.8502	Logistic	Exponential
		0.0217	0.0411	0.0661		
	MLP 5-11-5	0.9497	0.8688	0.8779	Logistic	Exponential
		0.0164	0.0393	0.0537		

**Table 5:** Correlation coefficients for ANN prediction of (i) total phenolic content (TPC) and antioxidant capacity, and (ii) content of individual selected phenolic compounds in ground ivy extracts based on NIR spectra.

Prediction	ANN Output	R^2^		
		Training	Test	Validation
Total phenolic content and antioxidant capacity	TPC	0.9207	0.9079	0.8928
	ABTS	0.8830	0.8864	0.8745
	DPPH	0.8974	0.8677	0.8315
Content of individual selected phenolic compounds	Chlorogenic acid	0.9545	0.9462	0.8826
	Cryptochlorogenic acid	0.9283	0.8982	0.8006
	Caffeic acid	0.9685	0.9613	0.8999
	Rosmarinic acid	0.9407	0.9300	0.8139
	Rutin	0.9442	0.9386	0.9049

## Data Availability

Not applicable.

## Supplementary Materials

The following supporting information can be downloaded at: https://www.mdpi.com/article/10.3390/foods11050658/s1, Figure S1: Geographical location of the ground ivy collection places, Figure S2: Sample of collected ground ivy (sample G3), Figure S3: HPLC chromatograms (recorded at 320 nm) of a) MAE extract and (b) SWE extract of sample G5, Figure S4: Fragmentation profile of the most dominate ion detected in F1, *m*/*z* 353, Figure S5: Fragmentation profile of the most dominate ion detected in F2, *m*/*z* 593, Figure S6: Fragmentation profile of the most dominate ion detected in F3, *m*/*z* 519, Figure S7: Fragmentation profile of the most dominate ion detected in F4, *m*/*z* 239.
