# Supplementary material for: Comprehensive Study of Traditional Plant Ground Ivy (Glechoma hederacea L.) Grown in Croatia in Terms of Nutritional and Bioactive Composition
Source: Foods. 2022 Feb 23;11(5):658. doi: 10.3390/foods11050658 (PMC8909519; doi:10.3390/foods11050658)
Supplement: Supplementary file 1 [file foods-11-00658-s001.zip › foods-1594474-supplementary.pdf]

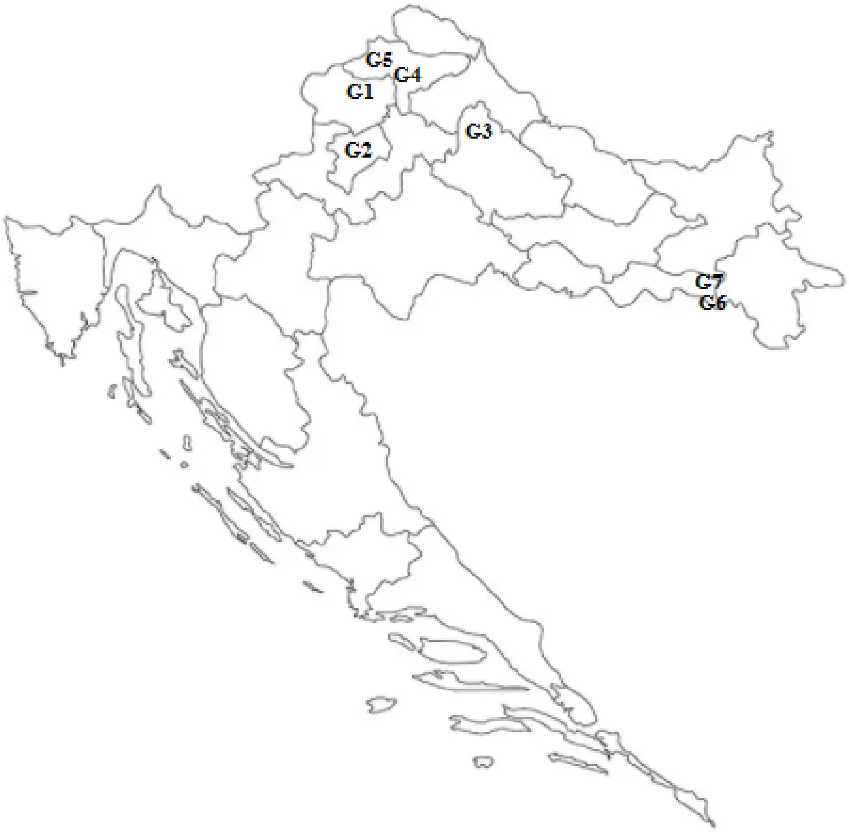

Figure S1. Geographical location of the ground ivy collection places

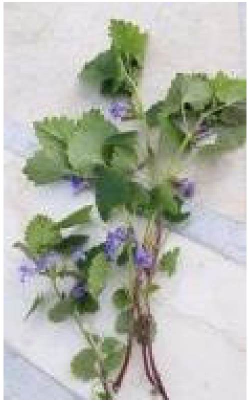

Figure S2. Sample of collected ground ivy (sample G3)

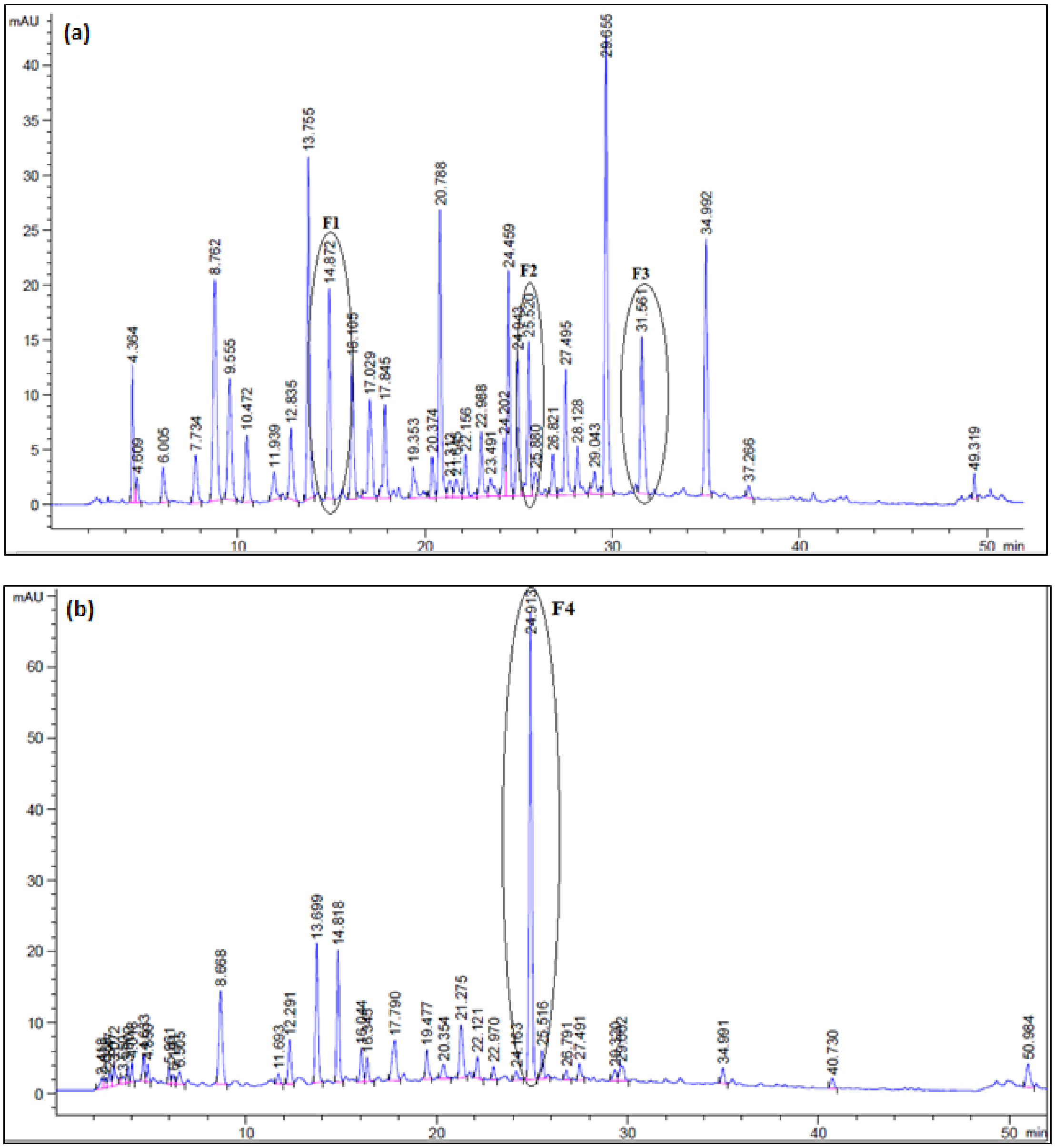

Figure S3. HPLC chromatograms (recorded at 320 nm) of a) MAE extract and (b) SWE extract of sample G5

MS/MS fragmentation profiles of unknown peaks from ground ivy

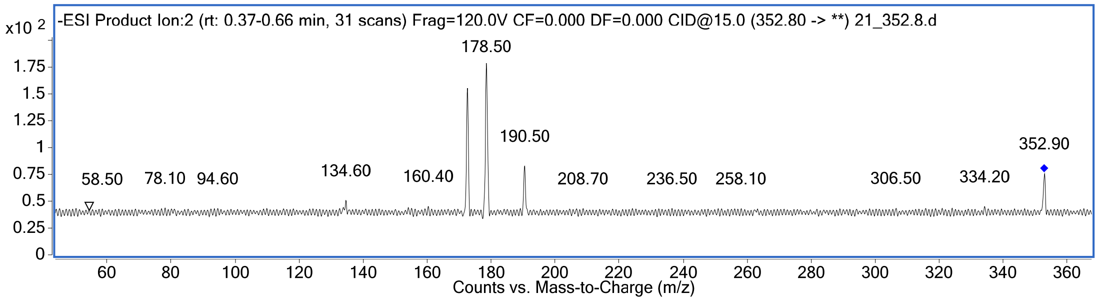

Figure S4. Fragmentation profile of the most dominate ion detected in F1, *m/z* 353

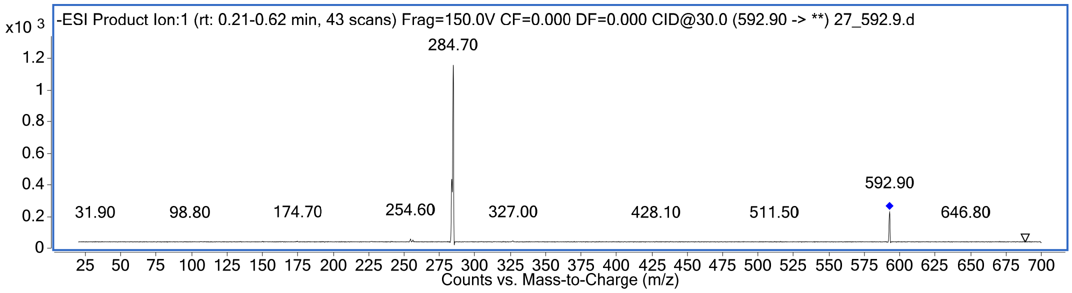

Figure S5. Fragmentation profile of the most dominate ion detected in F2, *m/z* 593

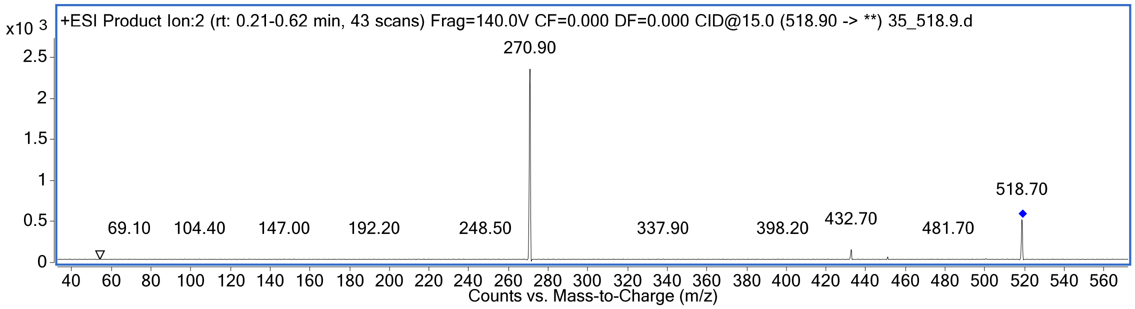

Figure S6. Fragmentation profile of the most dominate ion detected in F3, *m/z* 519

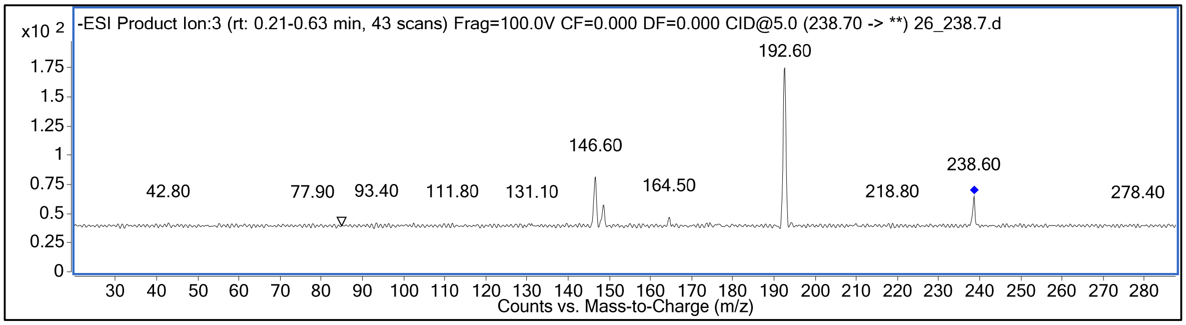

Figure S7. Fragmentation profile of the most dominate ion detected in F4, *m/z* 239
